# Contribution of Raman Spectroscopy to Diagnosis and Grading of Chondrogenic Tumors

**DOI:** 10.1038/s41598-020-58848-0

**Published:** 2020-02-07

**Authors:** Mario D’Acunto, Raffaele Gaeta, Rodolfo Capanna, Alessandro Franchi

**Affiliations:** 1 IBF-CNR, Istituto di Biofisica, Consiglio Nazionale delle Ricerche, Area della Ricerca di Pisa, via Moruzzi 1, I-56124 Pisa, Italy; 2 Department of Translational Research and of New Technologies in Medicine and Surgery, University of Pisa, Pisa, Italy

**Keywords:** Bone cancer, Cancer

## Abstract

In the last decade, Raman Spectroscopy has demonstrated to be a label-free and non-destructive optical spectroscopy able to improve diagnostic accuracy in cancer diagnosis. This is because Raman spectroscopic measurements can reveal a deep molecular understanding of the biochemical changes in cancer tissues in comparison with non-cancer tissues. In this pilot study, we apply Raman spectroscopy imaging to the diagnosis and grading of chondrogenic tumors, including enchondroma and chondrosarcomas of increasing histologic grades. The investigation included the analysis of areas of 50×50 μm^2^ to approximately 200×200 μm^2^, respectively. Multivariate statistical analysis, based on unsupervised (Principal Analysis Components) and supervised (Linear Discriminant Analysis) methods, differentiated between the various tumor samples, between cells and extracellular matrix, and between collagen and non-collagenous components. The results dealt out basic biochemical information on tumor progression giving the possibility to grade with certainty the malignant cartilaginous tumors under investigation. The basic processes revealed by Raman Spectroscopy are the progressive degrading of collagen type-II components, the formation of calcifications and the cell proliferation in tissues ranging from enchondroma to chondrosarcomas. This study highlights that Raman spectroscopy is particularly effective when cartilaginous tumors need to be subjected to histopathological analysis.

## Introduction

Cancer diagnosis remains one of the biggest challenges in medicine. The development of new noninvasive strategies or the improvements of existing ones makes Raman Spectroscopy (RS) fundamental for diagnosing the chemical compositions of cells and tissues. RS is able to probe fundamentals vibrational states of biomolecules, and exploits a label-free and non-destructive optical approach. RS is thus being used more and more frequently to analyses biological tissues^1–6^. In fact, for various types of cancers, *in vivo* biopsy imaging and histopathological analyses are carried out using RS^7–11^. RS is also exploited to evaluate the biochemical attributes of bones, and has revealed pathological changes in the components of the bone matrices. These changes include alterations in phosphate, carbonate and collagen degradation, as well as spectral changes in terms of bone metastasis primed by prostate and breast cancer^11–13^. With these abilities, the application of RS to the early diagnosis of bone tumors is more than ever necessary.

**Figure 1:**
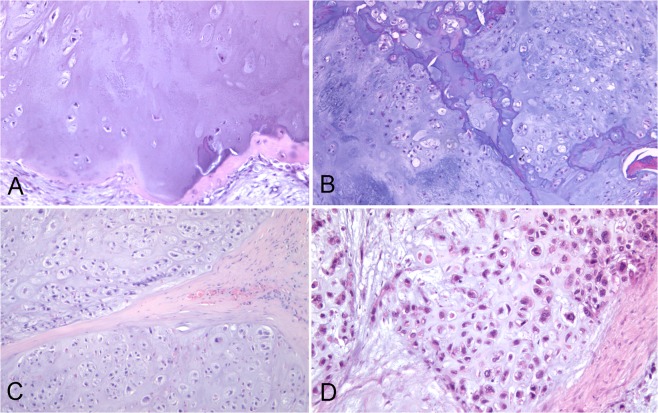
Representative histologic images of the tumors analyzed in this study (hematoxylin and eosin staining). (**A**) EC; (**B**) CS G1; (**C**) CS G2; (**D**) CS G3.

In the present pilot study, we apply the RS spectral imaging technique to improve non-destructive diagnosis and grading of chondrogenic tumors. Cartilaginous tumors are the most frequent primary bone tumors. While the true incidence of enchondromas (ECs) is difficult to determine because they are often asymptomatic, central chondrosarcoma (CS) accounts for approximately 20% of malignant bone tumors^14^, with an incidence, for example, of 8.78 per million inhabitants between 2005 and 2013 in the Netherlands^15^, whereas the overall rate incidence of CSs is estimated approximately to be 1 in 200,000 per year^16^, and it is the third most frequent malignant bone tumor after multiple myeloma and osteosarcoma engraving for approximately 20~30% of primary malignant bone tumors^17^. The clinical behavior of CSs is strictly related to the histologic grade, which is assigned according to the criteria established by Evans *et al*.^18,19^. Grade I CS (CS G1) metastasize only exceptionally, whereas in grade II CS and grade III CS (CS G2 and CS G3) the risk of metastasis is significantly higher (up to 70%). However, the differential diagnosis between EC and CS, as well as the grading of CSs remains a challenging task even for experienced bone tumor pathologists, with low consensus and reproducibility^20,21^, Fig. 1. Still, these distinctions are important to guide surgical management, which for the moment is the only effective form of treatment, given that cartilage tumors do not respond to conventional chemotherapy and radiotherapy. Currently, an intralesional treatment procedure or a wait-and-see approach are appropriate for ECs, while CS G1 of the appendicular skeleton with no aggressive imaging features may be treated more aggressively using curettage with local adjuvant therapy or by en bloc resection if they present radiologic signs of aggressiveness^22^. CS G2 and CS G3 are treated with en-bloc resection.

Our results evidenced the progressive collagen type-II (here and in the following, the term collagen will always indicate collagen type-II, unless otherwise indicated) degradation ranging from EC to CS G3 tissues. In addition, multivariate analysis revealed that the progressive modifications have a relevant change between CS G1 and CS G2, if compared to changes correspondent to EC- CS G1, or CS G2- CS G3, respectively. In turn, local biochemical modifications due to calcifications, Extracellular Matrix (ECM), or chondrocyte cells are clearly evidenced. Such findings have highlighted that Raman-based marker-free chemical imaging is a powerful tool to identify the distribution of cellular and matrix components in cartilaginous tissues.

## Results

RS analysis was carried out with a Raman imaging microscope (RIM). A total of 10 patients were analysed, who were being treated at our Institution, Azienda Ospedaliera Universitaria Pisana, Pisa. The experimental dataset for the 10 patients numbered consecutively from 1 to 10 is presented in Table 1, which highlights the distribution of four sample groups. Group EC, (cases 1–3), group CS G1 (cases 4–6), group CS G2 (cases 7–8) and group CS G3 (cases 9–10). Formalin fixed paraffin embedded tumor tissue sections were collected on glass slides and subsequently submitted to RS analysis after de-paraffination step (for details see Chondrogenic Tissues subsection in the Methods section).

**Table 1:** Summary of the clinico-pathologic features of the cases analyzed. Total number of maps: 40, for a total number of approximately 10^5^ spectra. ANED = alive with no evidence of disease.

case	age	sex (F/M)	diagnosis	location	clinical treatment	follow up	N. of tissues maps
1	45	M	EC	proximal femur	curettage	ANED	4
2	62	M	EC	distal femur	curettage	ANED	3
3	43	M	EC	proximal humerus	curettage	ANED	3
4	17	F	CS G1	scapula	resection	ANED	4
5	51	F	CS G1	proximal humerus	curettage	ANED	3
6	53	M	CS G1	VI rib	resection	ANED	5
7	28	M	CS G2	distal femur	resection	ANED	5
8	45	M	CS G2	proximal phalanx of second finger	amputation	ANED	4
9	33	F	CS G3	pelvis	resection	Local recurrence and lymph node metastasis at 6 months	5
10	50	M	CS G3	pelvis	resection	Local recurrence at 12 months; lung metastases at 13 months	4

## Biochemical Study

**Figure 2:**
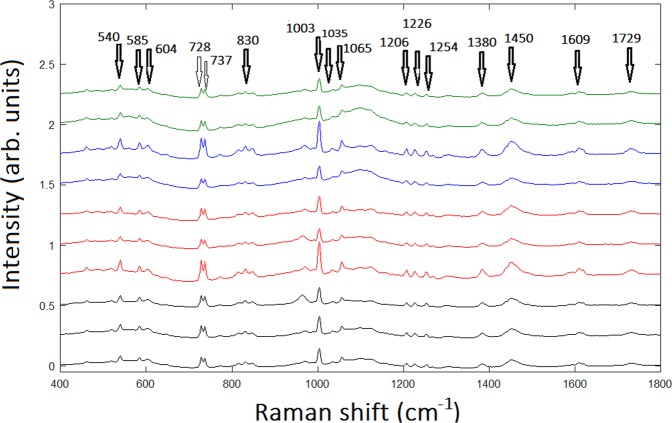
Averaged Raman spectra of 10 patients under investigation, EC, black line, CS G1, red line, CS G2, blue line, CS G3, green line, respectively. The arrows and the correspondent Raman shift value remark the most significant bands.

The spectra resulting from averaging the Raman maps were classified in relation to the different malignant tissue grades corresponding to 10 patients. In Fig. 2, the spectra correspondent to the 4 different groups for the 10 patients under investigation are displayed. One first evidence is that the spectra corresponding to the same CS grade were in close proximity despite belonging to different patients, thus denoting a remarkable homogeneity in the same grade of malignancy. Conversely, spectra corresponding to different grades present significant differences.

The assignation of Raman bands present in our spectra is made with the help of references^23–25^ and reported in Table 2. RS studies regarding cartilaginous tissues are recent^26,27^ while analogous Raman studies for CS are rather poor^28^. The most relevant differences regard the bands due to collagen (strong band at 1035 cm^−1^ present in EC, CS G1, CS G2, but absent in CS G3) and Proline (strong band at 1065–7 cm^−1^ present in EC, CS G1, CS G2, but absent in CS G3). Relevant strong Raman bands for collagen and Proline common to all tissues are at 728 cm^−1^ (very strong), at 830 cm^−1^ and at 1206 cm^−1^. Several common bands correspond to non-collagenous amminoacids such as Phenylalanine (Phe), Triptophan (Trp) or Tyrosine (Tyr), which are also efficient Raman scatterers. Common bands correspondent to chondroitin sulfate are revealed by a strong band at 1380 cm^−1^ of Glycosaminoglycan (GAG) and a medium band at 604 cm^−1^ of Glutamate. These results are not surprising, given that collagen and chondroitin sulfate are the primary components of cartilaginous tissues. GAGs consist of linear polysaccharide chains composed of repeating disaccharide units and form proteoglycans by covalently attaching to their core proteins. Chondroitin sulfate is a sulfated GAG with the disaccharide unit of beta-D-galactosamine (GalNAc) and beta-D-glucuronic acid (GlcA), and often modified with ester-linked sulfate at specific positions. A significant result highlighted by Table 2 is the band (strong intensity) at 1450 cm^−1^ assigned to CH_2_ bending mode in malignant tissues and evidenced only by CS G2 and CS G3.

**Table 2:** Peak position and assignment of major Raman bands observed in the tissues under analysis. Peaks intensity: w = weak, m = medium, s = strong, vs = very strong. Ref. ^23–25^.

Raman shift (cm^−1^)	Band assignment	EC	CS G1	CS G2	CS G3
428 (m)	Symmetric stretching of phosphate (HA)	√			
435 (w)	Pyruvate				√
454 (w)	Ring torsion phenyl				√
461 (m)	Glucosamine		√	√	√
481 (w)	DNA				√
486–90 (w)	Glycogen	√	√	√	√
499 (w)	Glutamate				√
509 (w)	S-S disulphide stretching band of collagen			√	√
519 (s)	Phosphatidylinositiol	√	√	√	√
527 (s)	N-Acetyl-D-Glucosamine		√		
540 (s)	ν(S-S) Cysteine	√	√	√	√
559 (w)	Coenzyme A	√			
585 (s)	OH out of plane bending	√	√	√	√
604 (m)	Glutamate	√	√	√	√
621 (w)	C-C twisting mode of Phe		√		
646 (w)	C-C twisting of Tyr	√			
728 (vs)	Proline (collagen assignment)	√	√	√	√
737 (vs)	Thymine (DNA)	√	√	√	√
776 (w)	Phosphatidylinositiol	√	√		
815 (m)	Proline	√	√	√	
830 (s)	Proline	√	√	√	√
847 (m)	C-O-C skeletal mode	√	√	√	
969–70	DNA	√	√	√	√
1003 (vs)	Phe	√	√	√	√
1035 (m)	Collagen	√	√	√	√
1056 (m)	Lipids	√	√	√	√
1065–7 (s)	Proline	√	√	√	√
1096 (m)	Phosphodioxy (PO_2_)^−^	√			
1106 (m)	Phe in proteins		√	√	
1123 (m)	C-N (protein assignment)	√	√		
1128–9 (m)	C-N (C-C) stretching				√
1170 (m)	Tyr				√
1206 (s)	difference in collagen content	√	√	√	√
1226 (s)	Amide III	√	√	√	√
1254 (s)	C-N in plane stretching	√	√	√	√
1268 (w)	Amide III (collagen assignment)	√	√		
1313 (m)	CH_3_CH_2_ twisting mode of lipids				√
1346 (m)	CH_3_CH_2_ wagging mode of lipids				√
1373 (m)	Ring breathing modes of DNA/RNA bases				√
1380 (s)	Glycosaminoglycans	√	√	√	√
1398 (w)	C = O symmetric stretching				√
1450 (s)	CH_2_ bending mode in malignant tissues			√	√
1485–8 (w)	Ring breathing modes in DNA				√
1588 (s)	Phe, hydroxyproline				√
1595 (s)	(CO_2_)^−^ antisymmetric stretching	√	√	√	√
1609 (m)	Cytosine (DNA)	√	√	√	√
1622 (m)	Trp				√
1729 (m)	C = O ester group	√	√	√	

Another significant result represented in Fig. 2 is that, in line with other studies on cartilage biochemical composition^29,30^, increasing of degradation of collagen is strictly connected to the decreasing of the whole Raman spectrum, although most of the representative collagen Raman bands are still present. Characteristic DNA bands present common peaks at 737 cm^−1^, very strong and corresponding to thymine, and medium intensity bands at 969–70 cm^−1^ and at 1609 cm^−1^ corresponding to cytosine. A medium intensity band at 1373 cm^−1^ corresponding to ring breathing modes of DNA/RNA bases is, on the contrary, present only in CS G3. Similarly, even the medium intensity bands at 1313 cm^−1^ and 1346 cm^−1^, assigned to CH_3_CH_2_ twisting mode of lipids and CH_3_CH_2_ wagging mode of lipids, respectively, indicate a greater cell proliferation, since the lipids constitute about 50% of the mass of the plasma membrane.

**Figure 3:**
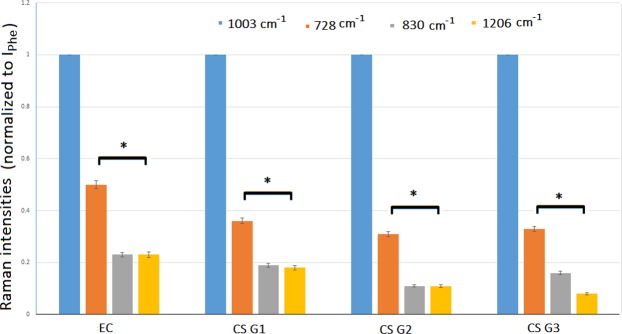
Comparison of the normalized intensities of Phe (I_Phe=1003_ = 1) and three selected bands assigned to collagen (I_728_, I_830_, I_1206_) for EC, CS G1, CS G2, CS G3, *p < 0.05.

The progressive degradation of collagen from EC to CS G3 was tested considering the ratio among three bands assigned to collagen (728 cm^−1^, 830 cm^−1^ and 1206 cm^−1^) to Phe, 1003 cm^−1^, this last band used to normalize the Raman intensity. Statistical significance was verified by analysis of variance, employing the Fisher’s Least Significant Difference test. Data with *p*-values that were lower than 0.05 were identified as statistically significant. Considering the band corresponding to Phe as the unity for all the samples, the results are reported in Fig. 3, we obtain a *p*-value = 0.018 (*F* = 5.71). The calculation was made using the Matlab function *anova2*.

**Figure 4:**
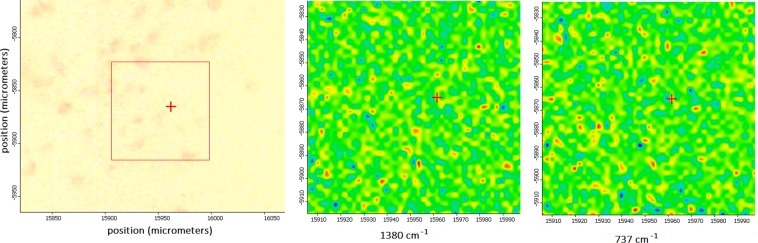
Raman maps for localization of GAG 1380 cm^−1^, image in the middle, and of DNA band at 737 cm^−1^, image on the right, in chondrocytes in CS G3 tissue, image on the left. The red-yellow spots denote higher concentration of GAGs and DNA band, respectively.

One interesting biochemical connection between chondroitin sulfate and DNA can be evidenced by using Raman maps obtained through the application of RIM to the sample tissues. Chondroitin sulfate has generally higher concentrations in the pericellular tissue, i.e., the portion of matrix immediately surrounding chondrocytes, whereas collagen is at its densest within the ECM. Our observation about this recognized connection is made in Fig. 4. We selected a portion of CS G3 tissue showing a certain amount of chondrocytes and then identified the areas denoting the high amount of bands assigned to DNA (737 cm^−1^ strong, and 1609 cm^−1^ medium) or GAG (1380 cm^−1^), this last as representative of chondroitin sulfate. In Fig. 4 the red-yellow spots denote the higher concentration of GAGs and thymine DNA in the cell nuclei, respectively.

**Figure 5:**
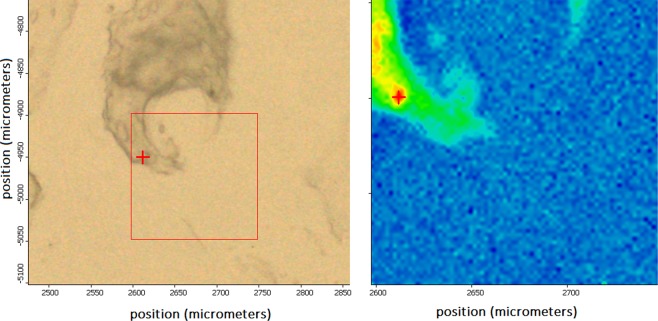
Identification of ossification process of calcifications in a sample of CS G1 with Raman band at 960 cm^−1^ correspondent to HA. On the left, the morphology image of a calcification; on the right the correspondent Raman hyperspectral map. The red-yellow spots denote higher concentration of HA.

In turn, topography analysis evidenced the formation of calcifications, a finding in agreement with the histologic appearance of cartilaginous tumors that frequently show amorphous calcifications and ossification. In some instances, extensive bone formation may lead to a misdiagnosis of osteosarcoma^19,31^. Hence, it is crucial to recognize this variant of ossification and bone formation identifying their specific biochemical components. This distinction has important consequences because of the different clinical behavior and management of these tumors. By applying RIM, the bony nature of the concretions is immediately identified by Raman band of phosphate group of Hydroxyapatite (HA) at 960 cm^−1^ ^32^, Fig. 5, as evidenced by Raman map.

## Statistical Analysis

The classification of the different chondrogenic tumor samples requires multivariate analysis. Principal Component Analysis (PCA) and Linear Discriminant Analysis (LDA) are very effective in differentiating between tissues, and between cells and ECM, and between collagen and non-collagenous components at a low computational cost.

PCA breaks down the spectral data generated by the measurements of a chemical system by reducing the size to a smaller number of principal components (PCs) that maximize the variance of our spectral data, see Statistical methods in Methods section. The data are classified by selecting various combinations of PCs with which to build a new coordinate system. LDA method works in a similar way to PCA. However, instead of identifying the component axes maximizing the variance of data as made by PCA method, LDA additionally finds the axes that maximize the separation between multiple classes, eventually previously identified by PCA, see Statistical methods in Methods section. Thanks to such peculiar features, PCA and LDA are widely used in Raman spectroscopy investigations for pathological classification^33^.

First, we used PCA to extract the relevant information from the original data by assessing thoses PCs that cover > 95% of the total variance of the raw data. To get the PCs, we built an *i* × *j* matrix of Raman spectra (where *i* indicates the number of spectra and *j* denotes the wave number) where the highest spectral differences can be associated with the PCs. In our study, the PCs were identified on a matrix 40 × 1400 of 40 averaged spectra including all several malignant degree of CS. Once first PCA procedure made it possible to reveal the most relevant differences among the CS samples, the second step, based on the main peaks previously was to apply LDA to find and classify any differences among the populations being investigated. The procedure revealed that the first five PCs contain all the most prominent information about any difference in chemical composition. These five PCs have a larger variance percentage, PC1(61,7%), PC2(15,9%), PC3(11,3%), PC4(4,2%) and PC5(2%), respectively, whose total variance is > 95%.

At this point, after checking the hypothesis of equality between the variances of two groups by Fisher’s test, we identified those PCs that differentiate those groups with benign (EC) tissues from those with malignant (CSs) tissues. A 2-sample *t*-test was used on the scores for each of the five PCs. The *t*-test confirmed that the 5 PCs are able to differentiate among CS tissues. In fact, the test showed the following *p*-values 0.027, 0.035, 0.041, 0.019, respectively, for the PCs.

**Figure 6:**
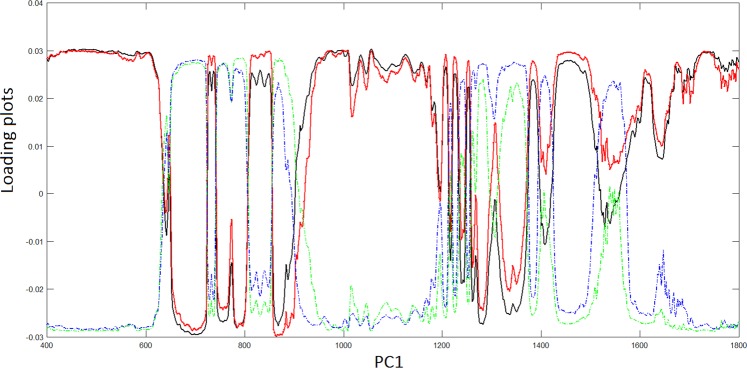
Loading plots of PC1 for the various CS grades. EC, black line; CS G1, rad line; CS G2, blue line; CS G3, green line.

For various dry cartilage zones, ECM is expected to be mainly constituted by collagen (approximately 50–60%), GAGs and proteoglycans (20–30%) along with non-collagenous proteins (10–20%)^26,34^. In principle, it could be possible to apply PCA to specific areas of tissue samples in order to discriminate ECM components. Bonifacio *et al*.^26^ showed that collagen components or DNA present negative or positive values of loadings, respectively. Our results show that PCA cannot discriminate between components belonging to collagen or DNA, respectively, because the most significant bands belonging to both collagen and DNA present generally positive values so making possible to apply PCA to discriminate between single ECM and cells components. However, the plots loading-PCs denote a remarkable sign inversion between the groups EC and CS G1 and the groups CS G2 and CS G3, respectively. In Fig. 6, we report the loading plot as a function of PC1 where we observe a minor variation between EC and CS G1 tissues and a change of sign with minor variations between CS G2 and CS G3 tissues, respectively. Similar results are obtained with the other PCs 2–5.

**Figure 7:**
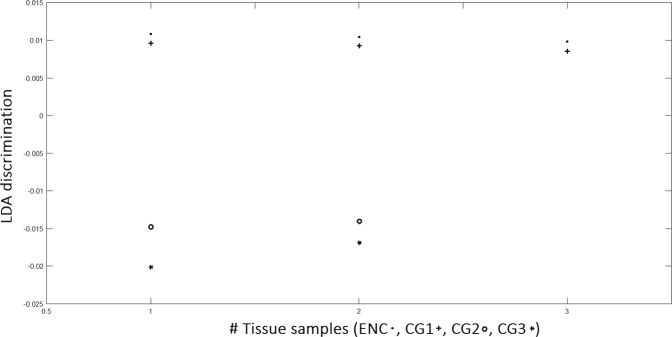
LDA analysis discrimination among the four tissue samples EC, CS G1, CS G2, CS G3.

**Figure 8:**
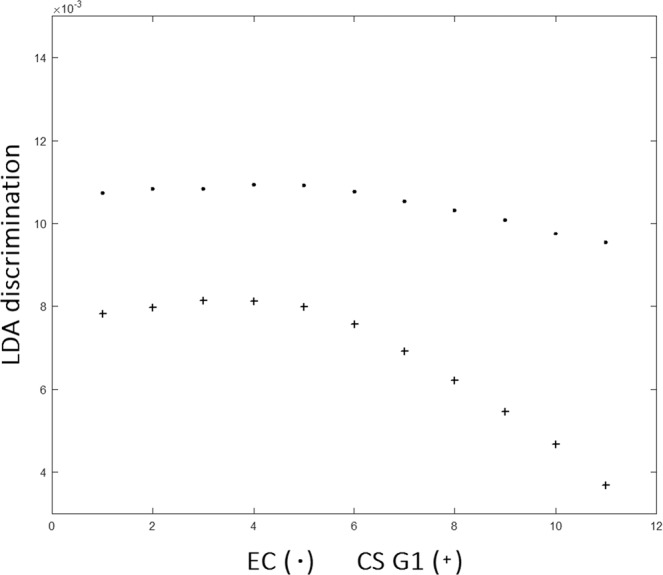
LDA analysis discrimination among averaged spectra belonging to EC or CS G1.

However, although PCA algorithm demonstrated some skills in discriminating the degree of diversity between the tissue typologies, denoting its diagnostic capacity, on the contrary, LDA algorithm has this full capability. A LDA model tested with a leave-one-patient-out cross validation was applied to the 40 × 5 matrix that resulted from the score of the 40 samples for the first five PCs, hence, the data are grouped for anyone of the four sample groups, see Statistical methods. The LDA was then applied to a linear combination of the PCs 1–5 giving about 100% of the cases correctly classified. The results for all the four sample groups are summarized in Fig. 6. It is noteworthy that the major differences are highlighted between the CS G1 and CS G2 samples resulting in a clinical low interobserver variability^35–37^. This is a relevant result because therapy for CS G1 and G2 may differ significantly. Grading of CSs is at present the best predictor of clinical behavior. CSs G1 are sparsely cellular, in contrast, CSs G3 are highly cellular. Up to 13% of recurrent CSs exhibit a higher grade of malignancy, thus suggesting that CS may biologically progress. Figure 7 confirms the extremely difficult distinction between EC and CS G1 resulting in high interobserver variability^38^. The results represented in Fig. 7 can complement histological examinations to guide clinical decision making. With regard to the critical issue with respect to a diagnosis relating to the CS G1, if we apply the LDA approach to spectra averaged relatively only to the EC and CS G1 groups, we obtain the results shown in Fig. 8. Differently to Fig. 7 where the scores are grouped relatively to any single patient, in Fig. 8 we report the scores regarding the averaged spectra belonging to EC or CS G1 groups. The scores reported in Fig. 8 show the ability to distinguish between EC and CS G1 spectra.

An additional training dataset of a higher number of spectra was employed to test the intraobserver variability looking at the sensitivity, specificity and accuracy of LDA model. In order to select a well-equilibrated new dataset, 15 spectra from grid sampling of hyperspectral images for each patient belonging to EC and CS G1 groups, respectively, and 20 spectra from grid sampling of hyperspectral images for any patient belonging to CS G2 and CS G3 group, respectively, for a total of 170 spectra. The LDA model was tested on such new dataset by the leave-one-patient-out cross validation and a test set, composed of a sub-set derived from the new dataset of 170 spectra was derived. For all the dataset and sub-sets taken in consideration, we have considered PCs covering > 95% of total variance of the data. In any case, we have obtained 5 PCs on which we built the linear expression for LDA, see Statistical Methods. For all the spectra dataset, we have generated LDA loadings as a function of patient groups, as in Fig. 6, finding a 90% of sensitivity, 90% specificity and 90% of accuracy between the spectra belonging to CS G1 and CS G2 groups, and approximately 75% of sensitivity, 70% specificity and 75% of accuracy for the spectra belonging to EC and CS G1 groups, and similar results for the groups CS G2, CS G3. Such results denote that the intraobserver variability of the hyperspectral images related to focus conditions, due to non-homogeneous topography of samples, and spectral noise did not influence the results.

## Discussion

In this paper, we applied RS to analyze the most relevant biochemical differences among cartilaginous tumors of bone from 10 patients. The RS approach providing biochemical information improves the diagnostic analysis based on morphological features. Currently, histologic grade remains the single best predictor of clinical behavior in CS. CS G1 behaves essentially as a locally aggressive tumor, with metastatic disease developing only in exceptional cases, whereas CS G2 and CS G3 present a high risk of distant metastases^18^^,^^39^. Our data indicating a greater difference in Raman spectra between CS G1 and CS G2 are in agreement with this shift in the biologic behavior of CS characterized by the acquisition of a metastatic behavior, thus underscoring their clinical relevance. The ability of RS to discriminate between the chondrogenic tumors grading with 90% of sensitivity, 90% specificity and 90% of accuracy by means of LDA algorithm has been evidenced. This ability of RS can have significant impact in the diagnosis of chondrogenic tumors.

Indeed, treatment of cartilaginous tumors is currently based on surgery, and consists of intralesional curettage for enchondroma, and of extensive intralesional curettage followed by local adjuvant treatment, such as phenolization or cryosurgery, in case of CS G1 of the limbs^22,38,40^. Wide, en-bloc excision is the preferred surgical treatment of CS G2 and CS G3, and of CS of flat bones. However, wide excision requires often major reconstruction procedures and can lead to considerable morbidity. Thus, histologic grading, and particularly the distinction between CS G1 and CS G2, is of outmost clinical importance not only for its prognostic value, but also for guiding treatment. Unfortunately, histologic grading of CS suffers of a high interobserver variability and low reproducibility^20,21^, and Raman spectroscopy could therefore complement conventional histopathology, helping to separate CS G1 from CS G2 or CS G3.

In the study of the whole spectrum of biological behavior of central cartilaginous tumors of bone, RS proved to be a fundamental tool to study tumor progression processes and grading. The progression of the grade of malignancy seems to be strongly correlated to several biochemical contents of ECM, such as degradation of collagen, cell proliferation or different biochemical composition of non-collagenous proteins in terms of proteoglycans contents. CSs are characterized by chondrocyte-derived hyaline-like ECM, which generally surrounds the tumor cells^37^, whereas hyaline-like ECM is composed of a woven network of collagen fibres and proteoglycans^34^. Raman analysis shows that one of the biomarkers indicating the modifications and degradation of collagen in cancerous tissues is Proline, one of the three amino acids forming the collagen α-helix. Proline metabolism plays a fundamental role in a number of regulatory targets in mammalian tissues and is particularly important in cancer^41^. Since Proline has specific regulatory functions an accurate understand of Proline role in cancer metabolism is highly desirable. These functions perform a leading role in apoptosis, autophagy and in response to nutrient and oxygen deprivation^41^. In addition, it is known that Pro-derived reaction oxygen species serves as a driving signal for cellular reprogramming. Additionally, in cartilaginous tissues, ECM, is formed principally by collagen, and collagen contains large amounts of Proline that may be metabolized to serve as a reservoir for Proline^41^. Thus, when cells are proliferating or not, they can store or not their metabolic substrates in the ECM^26,29^. However, when collagen is degrading, Proline is converted in amino acids^42^. This process is what has been highlighted by the RS, with bands assigned to Proline and collagen progressively less intense from EC to CS G1 and Cs G2, and the intensification of bands assigned to Tyr and Trp, as well as to the Phe for the CS G3. Non-collagenous proteins can be detected upon mapping the intensity of Raman bands associated with proteoglycans, such as chondroitin sulfate and GAG, or aromatic amino acids such as Phe, Tyr, and Trp, which are less present in collagen than in other protein^35–37^. To highlight the contribution from single cells, the images collected have a lateral resolution of 1μm, that is a compromise between yielding enough information about single cells and maximum lateral resolution achievable due to diffraction-limit of the radiation used to investigate the tissue samples.

Our combined histological and RS study provides a comprehensive grading of malignant cartilaginous tumors. The basic advantages of our approach are (i) a possible improved diagnostic efficacy, reducing the misclassification, (ii) a supplementary tool for medicine of precision and (iii) a relevant reduction of health costs.

The results have demonstrated that our method is highly cost-effective. This feature is possible because it essentially entails analysing unstained tissue sections. We believe our results can enhance the biochemical and imaging-based investigation of cartilaginous tissues, thereby triggering improvements in clinical applications and in diagnostic accuracy, and can also reduce inter-observer variability of chondrosarcoma tissues.

Given that few studies have reported RS investigations of cartilaginous and chondrosarcoma tissues, our results suggest that the different tumor grades can be unequivocally identified by RS. In addition, because underlying progressive collagen degradation, our next development of chondrosarcoma investigation will be to combine the RS with Brillouin spectroscopy^43,44^. As with RS, Brillouin signal is based on inelastic light scattering processes. In fact, whereas RS exploits high-frequency molecular rotational and vibrational modes, in Brillouin scattering the photons are scattered by low frequency phonons, thus providing information regarding viscoelastic properties. Consequently the combination of Raman and Brillouin spectroscopy, when applied to CS samples can provide label-free simultaneous information on elastic moduli and their biochemical composition, also in a 3D arrangement of tissues.

## Methods

### Ethics statement

The study was approved by the local Ethical Committee *Comitato Etico Regionale per la Sperimentazione Clinica della Regione Toscana, Sezione AREA VASTA NORD OVEST* (protocol number 14249). An informed consent was collected from all patients. For study participation of patients under the age of 18 years, a specific informed consent from a parent has been acquired (mod. C2; protocol number 14249). All the experiments were carried out in accordance with Good Clinical Practice (GCP) and with the ethical principles of the Declaration of Helsinki.

### Patients

Ten patients affected by primary chondrogenic tumors of the skeleton were enrolled in this study. Table 1 presents their salient clinico-pathologic features. All patients were diagnosed and treated at our Institution, Azienda Ospedaliera Universitaria Pisana, Pisa, in 2018.

### Chondrogenic tissues

The tumor tissue samples were prepared in line with current histopathological procedures. In each case, a formalin-fixed paraffin-embedded tissue block presenting only tumor tissue was selected. All specimens were undecalcified. Two sequential 5μm sections were obtained from each block and deposited on a glass slide. These were de-paraffinized, one was stained with hematoxylin and eosin, Fig. 1, and the other was left unstained and prepared for RS. One expert bone tumor pathologist (A.F.) evaluated the stained sections, and diagnosed and graded the tumors according to the criteria of the Current WHO Classification^19^.

### Raman spectroscopic measurements

**Figure 9:**
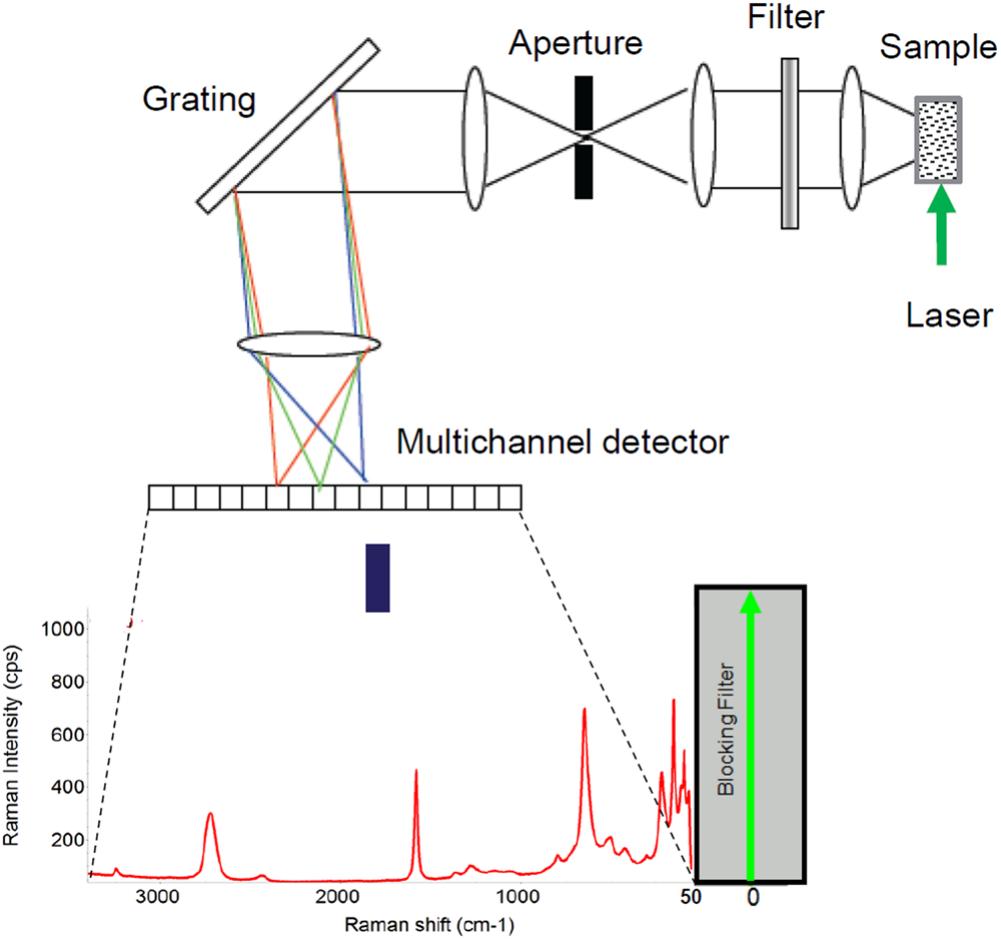
Schematic sketch of a DXR2xi Raman microscope equipped with a Rayleigh rejection filter with 50 cm^−1^ cut-off; a pinhole (confocal mode) aperture; a high resolution grating, 2 cm^−1^; and an electron multiplied CDD as detector.

A Thermo Fisher Scientific DXR2xi Raman microscope was used to record the Raman spectra, Fig. 9. An exhaustive description of the full functionality of a DXR2xi can be found in ref. ^45^. In our measurements, the configuration was with the following experimental parameters: laser wavelength 532 nm; power laser of 5–10 mW; 400–1800 cm^−1^ full range grating; 10×, 50× and 100× objectives; 25 μm confocal pinhole; 5(FWHM) cm^−1^ spectral resolution. Integration time for recording a Raman spectrum was 1 sec and 10 scans for any spectrum. As first step, the overview of tissue morphology in order to individuate the regions of interests was carried out with the collection of a number of mosaic images at low (10×) and intermediate (50×) magnification. Thus the acquisition of Raman spectra was carried out with a 100× objective. Optimization of signal-to-noise ratio and minimization of sample fluorescence were obtained through preliminary measurements in order to set the best experimental parameters. Multiple measurements were performed in different regions within the various samples, in order to assess intra-sample variability. In turn, no pre-treatment of the samples was necessary before Raman measurements. Peaks were identified with specific tool support by Omicron 9.0 software.

Raman hyperspectral chemical maps (briefly Raman maps) ranging from 50 × 50 μm^2^ (step size 1 μm) to approximately 200 × 200 μm^2^ (step size 4 μm), recording several hundreds of spectra per map were collected. One basic advantage of Raman maps is the localization of Raman spectra to specific areas so providing local information about chemical composition by selecting specific point by point. Step sizes were chosen to have a collection time for each map less than 7 hours for all the maps.

### Statistical methods

The data were processed making use of multivariate analysis. Two principal statistical procedures were used, an unsupervised method, PCA, and a supervised method, LDA^46^. A preliminary description is included in section Statistical analysis. PCA was firstly carried out with the purpose to reduce the initial high dimensionality of the dataset into the few latent variables, the principal components. Hyperspectral data were thus decomposed by PCA into latent spectra (loadings) and scores and visualized as in Fig. 6.

Thus, LDA was then applied on the same principal components previously observed in PCA to maximize the between class-separation. The goal of applying LDA was to find the feature subspace that optimizes the separability of the cartilaginous tissues analyzed. Briefly, LDA builds *j* = min(*k*−1,*p*) discriminant functions that evaluate discriminant scores (*S*_*ji*_) for each of *i* = 1,…,*n* subjects classified into *k* = 4 groups (EC, CS G1, CS G2, CS G3), from *p* = 5 linearly independent predictor variables (the PCs previously identified) as *S*_*ji*_ = *w*_*i1*_PC_*1i*_ + *w*_*i2*_PC_*2i*_ + … + w_*ip*_PC_*pi*_ [*i* = 1,…,*n* and *j* = 1,…,min(*k*−1,*p*)]. Hence, discriminant weights (*w*_*ij*_) are calculated by ordinary least squares. The estimation of such discriminant weight is such that the ratio of the variance within the k groups to the variance between the k groups is minimal. At this point, classification functions can be written as a linear combination of principal components and they are then of the type L_*ji*_ = *l*_*j0*_ + *l*_*j1*_PC_*1i*_ + *l*_*j2*_PC_*2i*_ + … + *l*_*jp*_PC_*pi*_ for each of the *j* = 1,…,*k* groups being constructed from the discriminant scores. It should be noted that in this approach, the coefficients of the classification functions for the *j*-th groups can be easily estimated from the within sum of squares matrices (*W*) of the discriminant scores for each group and from the vector of the PCs discriminant mean predictors in each of the classifying groups (*M*) according to the relation *L*_*j*_ = *W*^−1^*M* with *l*_*j0*_ = log_*p*−1/2_L_j_M_j_^46^.

In our LDA model, *Sensitivity* is the true positive rate: the proportions of actual positives correctly identified, i.e. it is the proportion of fraudulent classifications identified as fraudulent. Whereas *Specificity* is the true negative rate: the proportion of actual negatives correctly identified, i.e., the proportion of non-fraudulent classifications identified as non-fraudulent. In turn, *Accuracy* is the metric tool for evaluating the LDA classification model. Formally, *Accuracy* is the ratio between the number of correct predictions and the total number of predictions.

All computations were performed using customized software written in MATLAB version R2017b (Mathworks, Natick, MA, U.S.A.), cross-validation of customized software was made in some cases with R program, https://www.r-project.org/.

## Data Availability

The request for data sets, both raw and processed data, generated during the present study can be agreed and made directly to the corresponding author.
